# Stille’s Materia Medica

**Published:** 1874-12

**Authors:** 


### Bibliographical.

---

THERAPEUTICS AND MATERIA MEDICA. A Systematic Treatise on the Action and Uses of Medicinal Agents, including their description and history. By Alfred Stille, M.D., Professor of the Theory and Practice of Medicine and of Clinical Medicine in the University of Pennsylvania; Physician to St. Joseph's Hospital; Fellow of the College of Physicians, and Member of the Pathological Society of Philadelphia, etc., etc. Fourth edition, thoroughly revised and enlarged. In two volumes. Philadelphia: Henry C. Lea, 1874. Octavo; Leather; pp. 968 and 976. Price $12.

The new edition of Prof. Stille's work has undergone an entire revision. Many new articles have been introduced, the nomenclature conformed to the last edition of our Pharmacopœia, and two hundred and fifty pages added to accommodate the new matter. This edition retains the former classification, it being esteemed more natural and practical than any which has been more recently offered. "In the first edition of the work the author contended against the mischievous error of seeking to deduce the therapeutical uses of medicines from their physiological action. Continued study, observation and reflection have tended to strengthen his convictions upon this subject, and to confirm him in the faith that clinical experience is the only true and safe test of the virtues of medicines."

We make a few brief extracts to indicate the author's estimate of the efficiency of remedies in general: "Experience is really, as well as rationally, the only ground upon which curative effects can be expected from medicines. * * Medicine is only the handmaid of Nature, the really active and efficient agent in the restoration of health; medicine can do no more than remove the impediments from Nature's path, support her when faint, restrain her when violent, and guide her when she is inclined to err. * * The highest achievement of art is to sustain Nature, that she may have time and energy, if possible, to perfect her work. * * Between the two extreme classes above referred to—of diseases in which Nature alone is competent to the cure, and those in which neither Nature nor Art avails to avert death—there is a middle class, in which the skill of the physician and the power of his art are chiefly manifested. * * In these, medical skill alone outweighs the power of disease, and inclines the balance from death to life."

In looking over the work, we find no extreme views advocated upon any point; the author is moderate and conservative, as he should be, in the handling of all doctrines which are not well settled and fixed in the mind of the profession. There is an occasional looseness of expression, *e. g.*, in treating of syrup of iodide of iron—"the filtered solution is added to a hot *solution of syrup.*" We apprehend the meaning is hot syrup, or hot solution of sugar. Under the head of Calor we find no mention of the efficacy of water of intermediate temperature between the tepid bath and scalding water of blistering temperature—say at 150 to 170 F.—as a ready and prompt means of rallying comatose patients, in many conditions of opium poisoning, cerebral congestion, etc.

Proper caution is given against accident from the inhalation of chloroform, and the preference for ether as a general anæsthetic duly urged; but Nelaton's method of resuscitation, probably the best of all, is not mentioned. Under the head of Cathartic Enemata, the possibility of their passing the ileo-cæcal valve is rather grudgingly admitted, but no mention is made of the efficacy of the enema for overcoming intestinal obstructions of various kinds—to say nothing of the traversing of the entire alimentary canal with water from anus to mouth. We find also no mention of the modifying action of cane sugar upon the therapeutical properties of calomel, when the two are triturated together. All the new remedies of note find mention in their appropriate places.

The work we regard as a safe and excellent text book for the student, and guide to the practitioner. The volumes are neat, the print clear and comfortable to the eye, and the binding is solid and substantial, as it should be in a work of such frequent reference.

---

An Address, delivered before the McDowell Medical Society of Kentucky, at its Semi-annual Meeting, in Madisonville, Ky., Nov. 4th, 1874. By Wm. T. Briggs, M.D., of Nashville, Tenn. Published by order of the Society; 1874; octavo pamphlet; pp. 15.

